# Corneal Permeability and Uptake of Twenty-Five Drugs: Species Comparison and Quantitative Structure–Permeability Relationships

**DOI:** 10.3390/pharmaceutics15061646

**Published:** 2023-06-02

**Authors:** Cleildo P. Santana, Brock A. Matter, Madhoosudan A. Patil, Armando Silva-Cunha, Uday B. Kompella

**Affiliations:** 1 Department of Pharmaceutical Sciences, University of Colorado Anschutz Medical Campus, Aurora, CO 80045, USA; cleildopsantana@gmail.com (C.P.S.); brock.matter@cuanschutz.edu (B.A.M.); madhoosudan.patil@cuanschutz.edu (M.A.P.); 2 Faculty of Pharmacy, Federal University of Minas Gerais, Belo Horizonte 31270-901, MG, Brazil; armando@ufmg.br; 3 Department of Ophthalmology, University of Colorado Anschutz Medical Campus, Aurora, CO 80045, USA; 4 Department of Bioengineering, University of Colorado Anschutz Medical Campus, Aurora, CO 80045, USA; 5 Colorado Center for Nanomedicine and Nanosafety, University of Colorado Anschutz Medical Campus, Aurora, CO 80045, USA

**Keywords:** β-blockers, NSAIDs, corticosteroids, drug delivery, ocular delivery, drug permeability, QSPR, MLR

## Abstract

The purpose of this study was to determine corneal permeability and uptake in rabbit, porcine, and bovine corneas for twenty-five drugs using an N-in-1 (cassette) approach and relate these parameters to drug physicochemical properties and tissue thickness through quantitative structure permeability relationships (QSPRs). A twenty-five-drug cassette containing β-blockers, NSAIDs, and corticosteroids in solution at a micro-dose was exposed to the epithelial side of rabbit, porcine, or bovine corneas mounted in a diffusion chamber, and the corneal drug permeability and tissue uptake were monitored using an LC-MS/MS method. Data obtained were used to construct and evaluate over 46,000 quantitative structure–permeability (QSPR) models using multiple linear regression, and the best-fit models were cross-validated by Y-randomization. Drug permeability was generally higher in rabbit cornea and comparable between bovine and porcine corneas. Permeability differences between species could be explained in part by differences in corneal thickness. Corneal uptake between species correlated with a slope close to 1, indicating generally similar drug uptake per unit weight of tissue. A high correlation was observed between bovine, porcine, and rabbit corneas for permeability and between bovine and porcine corneas for uptake (R^2^ ≥ 0.94). MLR models indicated that drug characteristics such as lipophilicity (LogD), heteroatom ratio (HR), nitrogen ratio (NR), hydrogen bond acceptors (HBA), rotatable bonds (RB), index of refraction (IR), and tissue thickness (TT) are of great influence on drug permeability and uptake. When data for all species along with thickness as a parameter was used in MLR, the best fit equation for permeability was Log (% transport/cm^2^·s) = 0.441 LogD − 8.29 IR + 8.357 NR − 0.279 HBA − 3.833 TT + 10.432 (R^2^ = 0.826), and the best-fit equation for uptake was Log (%/g) = 0.387 LogD + 4.442 HR + 0.105 RB − 0.303 HBA − 2.235 TT + 1.422 (R^2^ = 0.750). Thus, it is feasible to explain corneal drug delivery in three species using a single equation.

## 1. Introduction

Ophthalmic drug products can be administered by systemic, periocular, intraocular, or topical routes [1,2]. The oral route, a noninvasive, systemic route, although convenient for dosing, is not a viable option for most ophthalmic drugs due to hepatic first-pass metabolism, extensive drug dilution in the blood, and the presence of blood–tissue barriers that limit ocular drug bioavailability. The blood–aqueous barrier, which limits drug transport from the systemic circulation to the anterior chamber [3], is constituted by the tight junctions of the ciliary non-pigmented epithelium and the endothelial layers of the iris and the inner wall of Schlemm’s canal. The blood–retina barrier, which limits drug transport from the systemic circulation to the retina [4], is constituted by the tight junctions of retinal endothelial cells (inner blood–retinal barrier) and retinal pigment epithelial cells (outer blood–retinal barrier). Both blood–tissue barriers are mainly imposed by tight junctions, with the blood–retinal barrier being more formidable, similar to the blood–brain barrier [4]. While the periocular and intraocular routes are invasive, the topical route allows noninvasive dosing to the eye.

The topical ophthalmic route, wherein the drug product is dosed noninvasively to the ocular surface, is widely used in treating eye diseases afflicting the anterior segment of the eye. Eye drops are the most commonly used topical ophthalmic drug products [5]. Although the eye is a readily accessible organ and eye drops are widely used, topical ocular drug delivery remains limited and challenging. This is due to a series of anatomical and physiological barriers of the eye, which can be broadly categorized as static or permeability barriers and dynamic or fluid flow barriers that limit drug delivery [6]. Static barriers for topical drug delivery to the anterior segment include the cornea and conjunctiva, especially tight junctions containing epithelial layers in these tissues. Dynamic barriers include blinking, nasolacrimal drainage, and blood and lymphatic flows. Additionally, metabolic barriers of eye tissues, including cytochrome P450 systems, proteases, and nucleases, may degrade topically applied drugs. The above barriers, in particular nasolacrimal drainage and epithelial permeability barriers, contribute the most towards low bioavailability of drugs to the anterior segment of the eye. In general, much less than 10% of the topically applied drug reaches intraocular tissues from an eye drop [7,8,9,10], and the bioavailability is predicted to be 1 to 5% for lipophilic drugs and less than 0.5% for hydrophilic drugs [11]. Currently, there are no FDA-approved eye drops to treat back-of-the-eye diseases due to inadequate delivery [5].

After topical administration of an eye drop in the precorneal area, it tends to accumulate in the conjunctival cul-de-sac and mix with the lacrimal fluid [3,4,12]. In the time interval between the administration and its complete drainage into the nasal cavity, the drop is expected to spread on the eye surface by the blinking movement [13]. The lacrimal fluid is composed of an external lipidic layer, a middle aqueous layer, and an internal mucin layer, and the components include electrolytes, lipids, proteins, and glycoproteins that may interact with drugs. The tear turnover time is about 1 to 2 min [14], and after this time, the majority of the drug administered can be lost through the nasal cavity or the conjunctiva into the systemic circulation [15].

The cornea is the main static barrier for drug absorption into the aqueous humor following topical administration. It is highly specialized, with three key regions: epithelium, stroma, and endothelium, which are lipophilic, hydrophilic, and lipophilic, respectively. The epithelium is composed of five layers of epithelial cells with tight junctions that form an important barrier to avoid fluid loss and pathogen penetration into the eye. Due to their highly lipophilic character, hydrophilic drugs show limited permeability across the corneal epithelium. Through this layer, drugs can permeate passively either between the cells via the paracellular pathway or through the cell or transcellular pathway. While the paracellular pathway prefers small hydrophilic drugs, the transcellular pathway prefers small lipophilic drugs. The corneal stroma is a highly hydrophilic layer that behaves like a liquid with a viscosity of about 1.5 times that of water for the diffusion of dextrans, allowing a permeability of dextrans as large as 34 nm [2]. Stroma is rate limiting for the transport of lipophilic drugs [11,16,17]. The endothelium, constituted by a leaky monolayer of cells, is less of a barrier than the epithelium. The corneal penetration of drugs is limited to compounds with a molecular weight typically lower than 500 Da; the average molecular weight of approved topical ophthalmic drugs is 392 Da, with a range of 111 to 1811 Da [18].

Aqueous solutions usually have the simplest manufacturing process and may result in high tissue concentrations since the soluble drug is at a molecular level and typically near the solubility limit of the drug. Corneal permeability is known to depend on drug properties such as lipophilicity, molecular size, charge, and shape [6,16,19,20,21] as well as formulation composition [9,22]. Understanding the factors affecting corneal permeability is expected to benefit the development of eye drops with enhanced bioavailability.

For the evaluation of corneal permeability of drugs, rabbit, porcine, and bovine corneas have been investigated [23,24], with rabbit being [23,25] the most commonly used animal model due to extensive ocular pharmacokinetic data availability [25]. The work herein employed the simultaneous analysis of 25 drug compounds of three different pharmacological classes using an innovative LC-MS/MS method to determine their tissue permeability and develop a predictive QSPR relationship of drug permeability in bovine, porcine, and rabbit corneas.

## 2. Materials and Methods

### 2.1. Chemicals

Alprenolol hydrochloride, atenolol, betaxolol hydrochloride, bromfenac sodium, calcium chloride, difluprednate, formic acid, flupirtine maleate, D-glucose, dimethyl sulfoxide (DMSO), HEPES (4-(2-hydroxyethyl)-1-piperazinethanesulfonic acid), indoprofen, ketoprofen, magnesium chloride, magnesium sulfate, mefenamic acid, methanol, metoprolol tartrate, nadolol, naproxen, nepafenac, pindolol, prednisolone, propranolol hydrochloride, sotalol hydrochloride, timolol maleate, tolmetin sodium salt dihydrate, and triamcinolone were purchased from Sigma-Aldrich, St. Louis, MO, USA. Acetonitrile, hydrochloric acid, potassium chloride, potassium phosphate dibasic, sodium chloride, and sodium phosphate dibasic were purchased from Fisher Scientific, Pittsburgh, PA, USA. Amfenac sodium monohydrate was purchased from VWR International LLC, Radnor, PA, USA. Sodium bicarbonate was purchased from Mallinckrodt Inc., Dublin, Leinster, Ireland. Budesonide, fluocinolone acetonide, and triamcinolone hexacetonide were purchased from Spectrum Chemical, New Brunswick, NJ, USA. Dexamethasone was purchased from Sigma-Aldrich, St. Louis, MO, USA, and Shanxi Jinjin Chemical Co., Ltd., Hejin, Shanxi, China. Oxprenolol hydrochloride was purchased from MP Biomedicals, Santa Ana, CA, USA. Dexamethasone-4,6,21,21-d4 was purchased from CDN Isotopes, QC, Canada. Flupirtine-d4 hydrochloride and timolol-d5 maleate were purchased from Santa Cruz Biotechnology, Santa Cruz, CA, USA.

### 2.2. Dosing Solution Preparation

The dosing solution was prepared from a drug cassette stock solution containing 25 drugs dissolved in DMSO at a final concentration of 200 ng μL^−1^. To prepare the dosing solution, 10 μL of the drug cassette stock solution was diluted to 10 mL in assay buffer (NaCl 122.0 mM, NaHCO3 25.0 mM, MgSO4 1.2 mM, K2HPO4 0.4 mM, CaCl2 1.4 mM, HEPES 10.0 mM and glucose 10.0 mM in water) (pH = 7.4) to achieve a final concentration of 200 ng mL^−1^.

### 2.3. Tissue Preparation

Bovine and porcine corneas were collected from freshly excised eyes purchased from local abattoirs (Elizabeth Locker Plant, Elizabeth, CO, USA). Freshly isolated rabbit corneas were shipped overnight from Pel-Freez (Rogers, AR, USA). The eyes were immersed in refrigerated HBSS solution during transport to the laboratory. The corneas were isolated from the eyes and mounted in NaviCyte Vertical Ussing chambers (San Diego, CA, USA), with their epithelial side facing the donor compartment.

### 2.4. Test Conditions

The dosing solution (1.5 mL) was placed in the donor side of the chambers, while plain assay buffer (1.5 mL) was placed in the acceptor side of the chambers. Both solutions and chambers were kept at 37 °C during the duration of the experiment (6 h). The aeration of the medium was performed using 5% CO_2_ and 95% air with slow bubbling. At 15, 30, 60, 120, 180, 240, 300, and 360 min, 50 μL samples were withdrawn from the acceptor side of the chambers, with immediate replacement of the volume with fresh assay buffer. Aliquots of the donor solution were also withdrawn at the end of the study from the donor side of the chambers. All samples were spiked with the internal standard solution immediately after collection.

### 2.5. Permeability Evaluation

The drug amount in the samples was quantified using LC-MS/MS, with sample data corrected for acceptor solution replenishment. Drug amounts in all samples were normalized as percent values of the initial amount of drug quantified in the dosing solution. The permeability of the drugs was compared using the apparent permeability coefficient [26] (*P_app_*), given by Equation (1):

(1) $$P_{app}=\frac{(dm/dt)\cdot V_{0}}{A\cdot m_{0}}$$

where *dm*/*dt* is the derivative of the cumulative drug amount transported through the tissue as a function of time, *V*_0_ is the volume on the acceptor side, *A* is the contact area of the tissue with the solutions, and m_0_ is the initial drug amount in the donor solution.

Permeability can be related to diffusivity [26] (Equation (2)) as follows:

(2) $$D=\frac{P_{app}\cdot h}{k}$$

where *P_app_* is the apparent permeability coefficient, *h* is the tissue thickness, and *k* is the partition coefficient of the drug between the tissue and dosing solution. Tissue thickness was calculated from results reported in the literature by different groups for bovine, porcine, and rabbit corneas (Table 1).

Drug flux [39] across the tissues was also used to compare tissue permeability (Equation (3)):

(3) $$J=\frac{dm/dt}{A}$$

where *dm*/*dt* is the derivative of the cumulative drug amount transported through the tissue as a function of time, and *A* is the contact area of the tissue with the solutions.

### 2.6. Drug Extraction

To extract the drugs from the cornea, the method developed by Matter, Bourne, and Kompella [40] was used. At the end of the experiment, the portion of the cornea exposed to the donor and acceptor solutions was taken from the chambers and submitted to a liquid–liquid extraction protocol before LC-MS/MS analysis. First, 50 ± 5 mg of each tissue was placed into fresh tubes. Briefly, 100 μL of fresh PBS and 10 μL of the internal standard were added to the tubes, and the content was homogenized using a glass-glass homogenizer. The samples were subjected to three freeze–thaw cycles, using liquid nitrogen (−196 °C) to freeze followed by room temperature thawing. Then, 300 μL of a solution of methanol and acetonitrile (2:1) was added to each tube, and after 30 min at room temperature, the samples were vortexed for 10 min and sonicated for 5 min. Afterwards, the tubes were centrifuged for 10 min, and the supernatant was collected into fresh tubes. The samples were evaporated (Savant SpeedVac SC100, Holbrook, NY, USA) until the sample volumes were less than 100 μL.

### 2.7. LC-MS/MS Analysis

For drug quantification, the method developed by Matter, Bourne, and Kompella [40] was used. An AB Sciex Qtrap 4500/Shimadzu HPLC was used, and the mobile phase consisted of a mixture of 0.1% formic acid in water (A) and 0.1% formic acid in 9:1 acetonitrile:water (B). The gradient elution was performed at 40 °C as follows: 99.4% of A (0–2 min), 94% of A (2.5 min), 47% of A (10.5 min), 6% of A (14 min), and 99.4% of A (14.5–18 min) using a Phenomenex Kinetex column. In the MS detector, the following transitions were monitored: 272.9/255 (sotalol), 267/190 (atenolol), 249/116 (pindolol), 309.9/254 (nadolol), 316.9/261 (timolol), 267.9/116 (metoprolol), 265.9/116 (oxprenolol), 304.9/196 (flupirtine), 395/375.1 (triamcinolone), 259.9/116 (propranolol), 249.9/116 (alprenolol), 308/116 (betaxolol), 361/343.1 (prednisolone), 254.8/238 (nepafenac), 393/373.2 (dexamethasone), 281.9/236 (indoprofen), 256/210 (amfenac), 453/433.2 (fluocinolone acetonide), 257.8/119 (tolmetin), 254.9/209 (ketoprofen), 231/185.1 (naproxen), 431/413.1 (budesonide), 333.8/316 (bromfenac), 509.1/303.1 (difluprednate), 241.9/224 (mefenamic acid) and 533.2/415.1 (triamcinolone hexacetonide). The equipment was operated at 500 °C with a spray voltage of 5 k and curtain and ion source gas pressures of 45 and 50 psi, respectively, along with an entrance and collision cell exit potential of 10 and 14 V, respectively.

### 2.8. Multiple Linear Regression (MLR) Modelling

LC-MS/MS quantification data were used to obtain predictive linear models of cumulative transport, permeability, and drug uptake for each tissue. Multiple linear regressions were performed using the least squares method with Microsoft Excel^®^ Professional 2016. The following molecular descriptors were calculated for each drug with ACDLabs^®^ (version 2019) and used as independent variables (Supplementary Materials Table S1): molecular weight, number of hydrogen bond donors, number of hydrogen bond acceptors, number of hydrogen bond donors and acceptors, total polar surface area, number of rotatable bonds, carbon ratio, nitrogen ratio, nitric oxide ratio, heteroatom ratio, halogen ratio, number of rings, number of aromatic rings, number of 5 atom rings, number of non-aromatic 6 atom rings, log(BCF), parachor, index of refraction, surface tension, density, polarizability, molar volume, molecular volume, molar refractivity, LogS, LogP, and LogD at pH of 7.2, 7.3, 7.4, 7.5, and 7.6.

**Figure 1:**
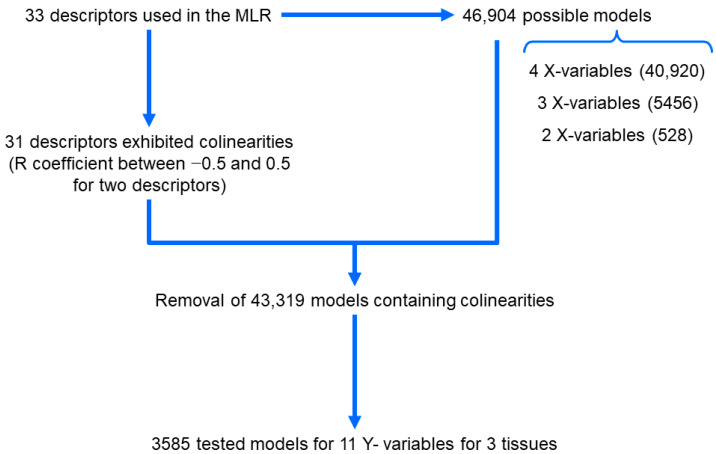
Rationale for the obtaining of MLR models.

To select the best-fit models, all possible collinearity-free models with four, three, and two independent variables were obtained, as described in Figure 1.

Only models presenting significant (*p* < 0.05) coefficients for all independent variables were selected and evaluated by R^2^, adjusted R^2^, and F values.

Best-fit models for each of the three parameters evaluated were submitted to internal cross-validation using the Q^2^ coefficient. Once the applicability domain of the models was defined [41], the Q^2^ coefficient was obtained with Microsoft Excel^®^ by sample splitting, where ≈20% of the samples constituted the test set. The splitting was repeated 1000 times for each model. The coefficient calculation followed the relationship [42] described by Equation (4):

(4) $$Q^{2}=1-\frac{\left[\sum_{i=1}^{test}({ŷ}_{i}-y_{i}{)}^{2}\right]/n_{test}}{\left[\sum_{i=1}^{tr}({ŷ}_{i}-{\bar{y}}_{tr}{)}^{2}\right]/n_{tr}}$$

where *n_test_* and *n_tr_* refer to the number of samples in the test and training sets, respectively, *y* represents the experimental value, ŷ represents the predicted value, and $\bar{y}$ represents the average value.

### 2.9. Bioavailability Estimation

Systemic and aqueous humor bioavailability values were calculated based on apparent permeability coefficients measured in the porcine cornea in the present study and those estimated for porcine conjunctiva. The conjunctival permeability was estimated using regression models and equations developed for each drug class based on a correlation of corneal and conjunctival permeability values reported in the literature [24,43]. In this step, only experimental articles simultaneously reporting data from porcine cornea and conjunctiva were considered.

*P_app_* values through porcine cornea or conjunctiva were each used to calculate the clearance through human tissues by multiplying the values with the human corneal (1.04 cm^2^) or conjunctival (17.64 cm^2^) total surface areas, unlike a prior study reporting the following equations, which used half of the conjunctival surface area [43]. From cornea and conjunctiva, the clearance (µL min^−1^) into human aqueous humor and into human systemic circulation were calculated, respectively. These values were then used to calculate bioavailability using the following relationships [43]:

(5) $$CL_{Topical}=CL_{Cornea}+CL_{Conjunctiva}+Q_{Tear}$$

(6) $$BA_{Aqueous}=\frac{CL_{Cornea}}{CL_{Topical}}\cdot 100$$

(7) $$BA_{Systemic}=\frac{CL_{Conjunctiva}}{CL_{Topical}}\cdot 100$$

where *CL_Topical_* represents the total clearance through the ocular surface, *CL_Cornea_* represents the clearance through cornea, *CL_Conjunctiva_* represents the clearance through conjunctiva, *Q_Tear_* represents the human tear flow rate (1.2 µL min^−1^) and the associated clearance [44], *BA_Aqueous_* represents the dose bioavailability in aqueous humor, and *BA_Systemic_* represents the dose bioavailability in systemic circulation.

### 2.10. Statistical Analysis

All the values are described as the mean ± standard deviation. Comparisons between groups were performed using a one-way ANOVA with GraphPad Prism 5.04, considering α = 0.05.

## 3. Results

### 3.1. Cumulative Transport and Permeability Coefficient

**Figure 2:**
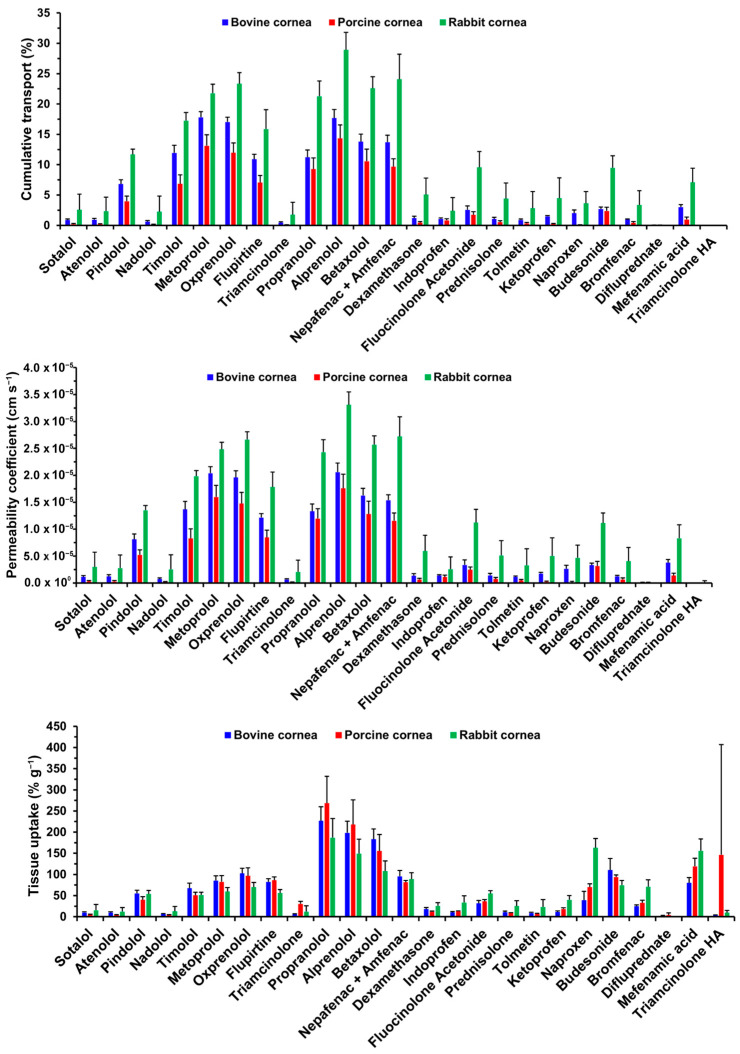
Cumulative transport, apparent permeability coefficient, and tissue uptake values. Mean ± STD is reported for 5 corneas.

The values of cumulative transport found for the drugs evaluated are presented in Figure 2.

The β-blockers were the drug class that showed the largest extent of transport across all corneas. For bovine cornea, the extent of transport was given as metoprolol, oxprenolol, and alprenolol > betaxolol > timolol and propranolol > pindolol > atenolol, nadolol, and sotalol. The drug extent of transport across porcine cornea was lower than that of bovine cornea, with alprenolol, metoprolol, and oxprenolol > betaxolol and propranolol > timolol and pindolol > sotalol, atenolol, and nadolol. Rabbit cornea showed a higher extent of β-blocker transport, with alprenolol > metoprolol, oxprenolol, propranolol, and betaxolol > timolol > pindolol > sotalol, atenolol, and nadolol. No significant differences were detected for sotalol, atenolol, or nadolol between the tissues, nor for pindolol between bovine and porcine corneas.

For the steroids, the extent of transport through the bovine cornea was budesonide and fluocinolone acetonide > dexamethasone and prednisolone > triamcinolone and difluprednate. In porcine tissue, the rank order was budesonide and fluocinolone acetonide > triamcinolone, dexamethasone, prednisolone, and difluprednate. In rabbit cornea, the extent of transport was higher than that of bovine and porcine tissues, with the rank order being fluocinolone acetonide and budesonide > triamcinolone, dexamethasone, and prednisolone > triamcinolone hexacetonide. No transport was detected for triamcinolone hexacetonide through bovine or porcine corneas or for difluprednate through rabbit cornea. Bovine and porcine corneas showed comparable results for all steroids. On the other hand, both tissues showed significantly lower transport of dexamethasone, fluocinolone acetonide, prednisolone, and budesonide than that observed in rabbit cornea.

In bovine cornea, the NSAIDs showed the extent of transport as nepafenac > flupirtine > mefenamic acid > indoprofen, tolmetin, ketoprofen, naproxen, and bromfenac. Porcine cornea showed the extent of transport as nepafenac > flupirtine > indoprofen, tolmetin, ketoprofen, naproxen, bromfenac, and mefenamic acid, with the overall extent lower than half of that for bovine. Rabbit cornea showed a higher extent of transport than that of bovine tissue, observed as nepafenac > flupirtine > indoprofen, tolmetin, ketoprofen, naproxen, bromfenac, and mefenamic acid. Flupirtine and nepafenac transport through porcine cornea was significantly lower than through bovine cornea, and both were lower than those observed for rabbit cornea. Mefenamic acid transport was comparable between bovine and porcine corneas but significantly lower than that observed for rabbit cornea.

Permeability coefficients for all drugs followed the same trends observed for cumulative transport, including significant differences. Data are presented in Figure 2. Therefore, differences between *P_app_* of drugs will not be described here to avoid redundancy.

### 3.2. Tissue Uptake

The tissue uptake data is presented in Figure 2.

Besides cumulative transport, β-blockers showed high uptake by the tissues. In bovine cornea, the amounts detected were higher for propranolol, alprenolol, and betaxolol > pindolol, timolol, metoprolol, and oxprenolol > sotalol, atenolol, and nadolol. In porcine and rabbit corneas, the extent of β-blocker uptake was propranolol > alprenolol and betaxolol > pindolol, timolol, metoprolol, and oxprenolol > sotalol, atenolol, and nadolol. The extent of β-blocker uptake was comparable between bovine and porcine corneas but significantly lower for propranolol, alprenolol, and betaxolol in rabbit cornea.

The extent of steroid uptake by bovine cornea was observed as budesonide > all the other steroids. In porcine cornea, steroid uptake was observed as budesonide > fluocinolone acetonide > triamcinolone, dexamethasone, prednisolone, difluprednate, and triamcinolone hexacetonide. Rabbit cornea showed an overall higher extent of steroid uptake, with budesonide > fluocinolone acetonide > triamcinolone, dexamethasone, and prednisolone > triamcinolone hexacetonide. No tissue uptake was quantifiable for difluprednate in rabbit cornea. Bovine and porcine corneas showed comparable extents of steroid uptake. Rabbit cornea showed higher fluocinolone acetonide uptake than bovine cornea and, on the other hand, lower budesonide uptake than bovine and porcine corneas.

Regarding bovine cornea, the extent of uptake of NSAIDs was nepafenac and mefenamic acid > flupirtine and naproxen > indoprofen, tolmetin, ketoprofen, and bromfenac. In porcine cornea, the extent of uptake of NSAIDs was in the order: mefenamic acid > flupirtine, nepafenac, and naproxen > indoprofen, tolmetin, ketoprofen, and bromfenac. Rabbit cornea showed a higher extent of uptake for the NSAIDs, with naproxen and mefenamic acid > nepafenac and bromfenac > flupirtine, indoprofen, tolmetin, and ketoprofen. Rabbit cornea showed higher uptake of naproxen and bromfenac than porcine cornea, which in turn showed higher uptake of naproxen and mefenamic acid than bovine cornea. Rabbit cornea showed higher uptake compared to bovine cornea regarding indoprofen, ketoprofen, naproxen, bromfenac, and mefenamic acid.

### 3.3. Correlation Plots

**Figure 3:**
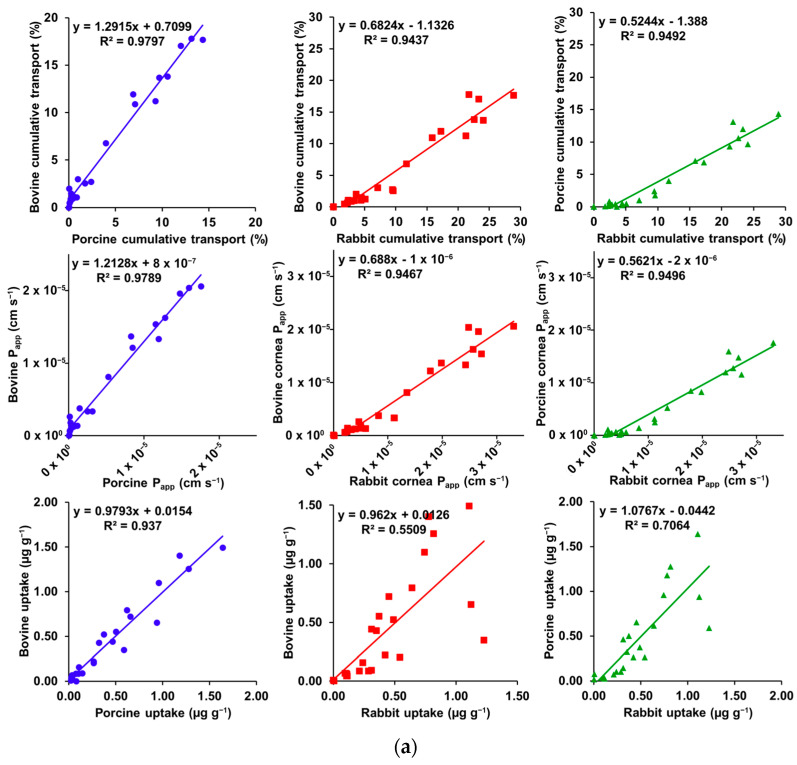

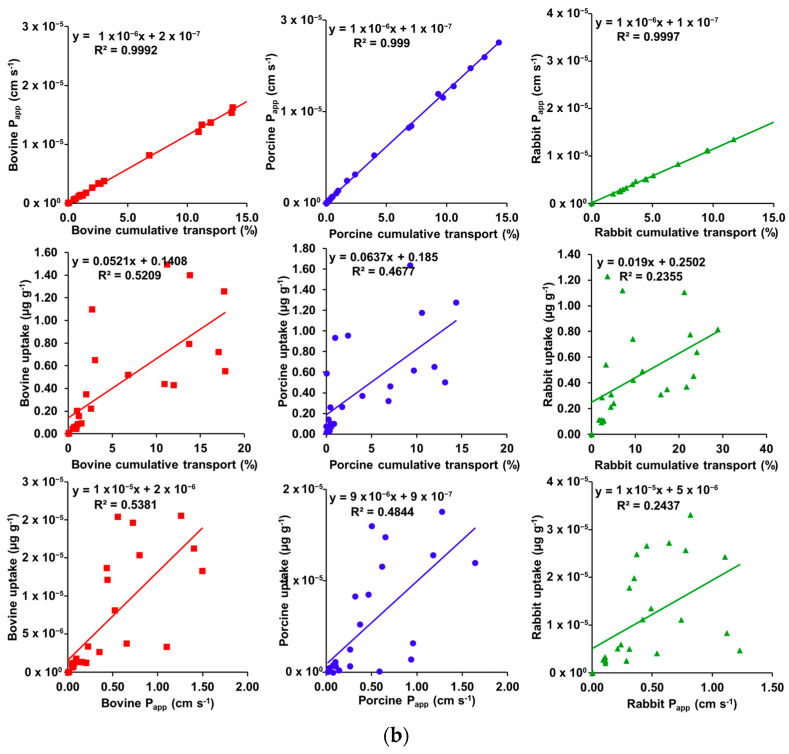
(**a**) Inter-species correlation plots for cumulative transport, *P_app_*, and tissue uptake. (**b**) Within species correlations for *P_app_* vs. cumulative transport, tissue uptake vs. cumulative transport, and tissue uptake vs. *P_app_*.

Various correlations were verified by R^2^ determination, with the graphs and results presented in Figure 3a,b.

Bovine vs. porcine cornea showed a high correlation for cumulative transport (R^2^ = 0.9797), *P_app_* (R^2^ = 0.9789), and tissue uptake (R^2^ = 0.9370), where the first two bovine corneas showed higher values and the last, lower values. When correlated to rabbit cornea, bovine and porcine corneas showed a high correlation for cumulative transport (R^2^ = 0.9437 and 0.9492, respectively) and for *P_app_* (R^2^ = 0.9467 and 0.9496, respectively), with both measurements showing higher values in rabbit cornea. However, bovine and porcine corneas showed weak or good correlation, respectively, with rabbit cornea for tissue uptake (R^2^ = 0.5509 and 0.7064, respectively).

Tissue uptake did not show a high correlation with *P_app_* or cumulative transport in bovine (R^2^ = 0.5381 and 0.5209, respectively), porcine (R^2^ = 0.4844 and 0.4677, respectively), or rabbit (R^2^ = 0.2437 and 0.2355, respectively) corneas. On the other hand, a high correlation (R^2^ ≥ 0.9990) was observed between *P_app_* and cumulative transport for all tissues.

**Figure 4:**
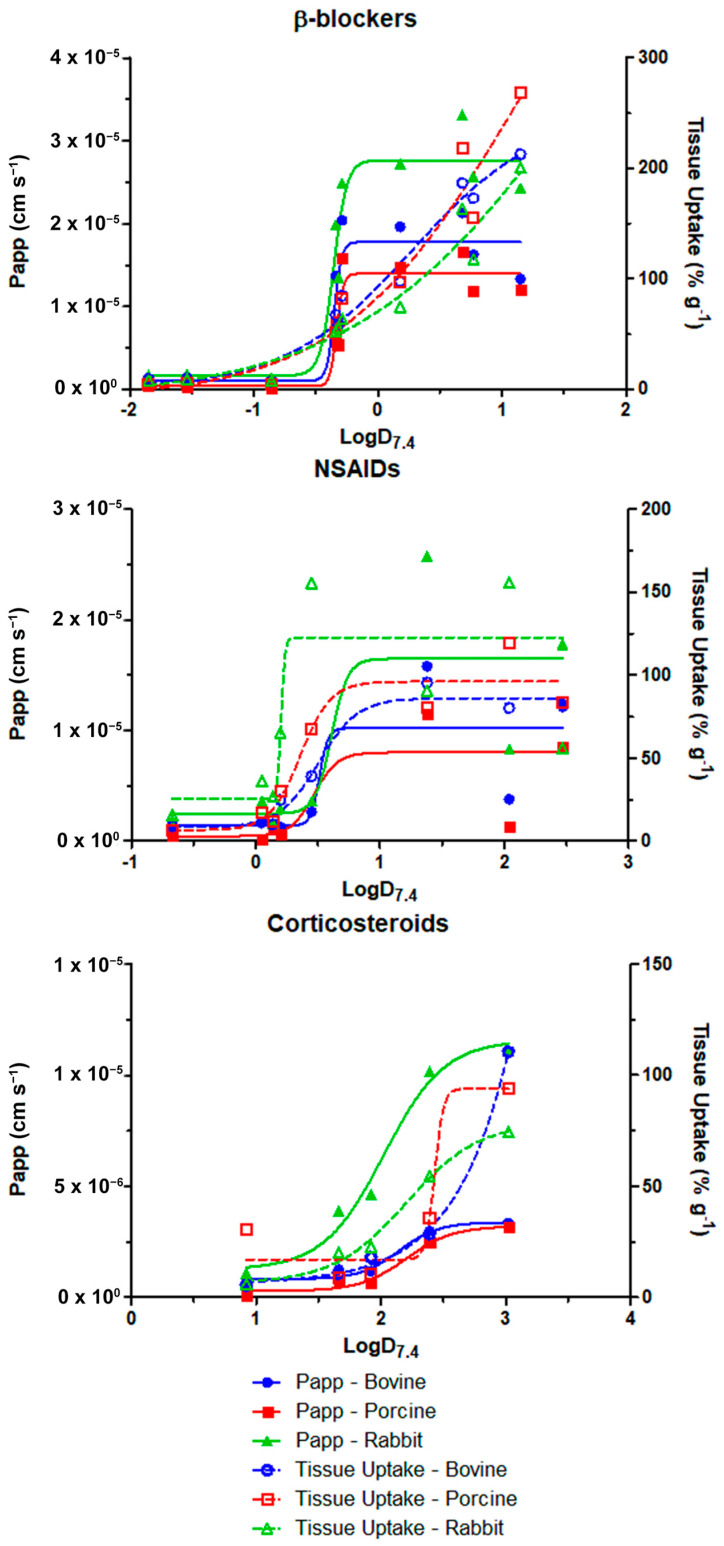
Correlation plots of permeability and tissue uptake with LogD at pH 7.4.

The permeability and tissue uptake also showed good correlation with LogD at pH 7.4, following sigmoidal relationships, which are presented in Figure 4.

To compare the behavior of the sigmoidal curves, Table 2 presents LogD_7.4_ values at half-maximum for each parameter:

### 3.4. MLR Modeling

The best-fit models obtained were ranked considering R^2^ and Q^2^ coefficients, and the models with the best performance for each tissue are described in Table 3:

Corneal thickness was considered a parameter to include the structure of the tissue as an x-variable in modeling. Results showed that considering the nature of the tissue in regression caused the resulting models to present higher robustness, as presented in Table 4.

### 3.5. Bioavailability Estimation

**Figure 5:**
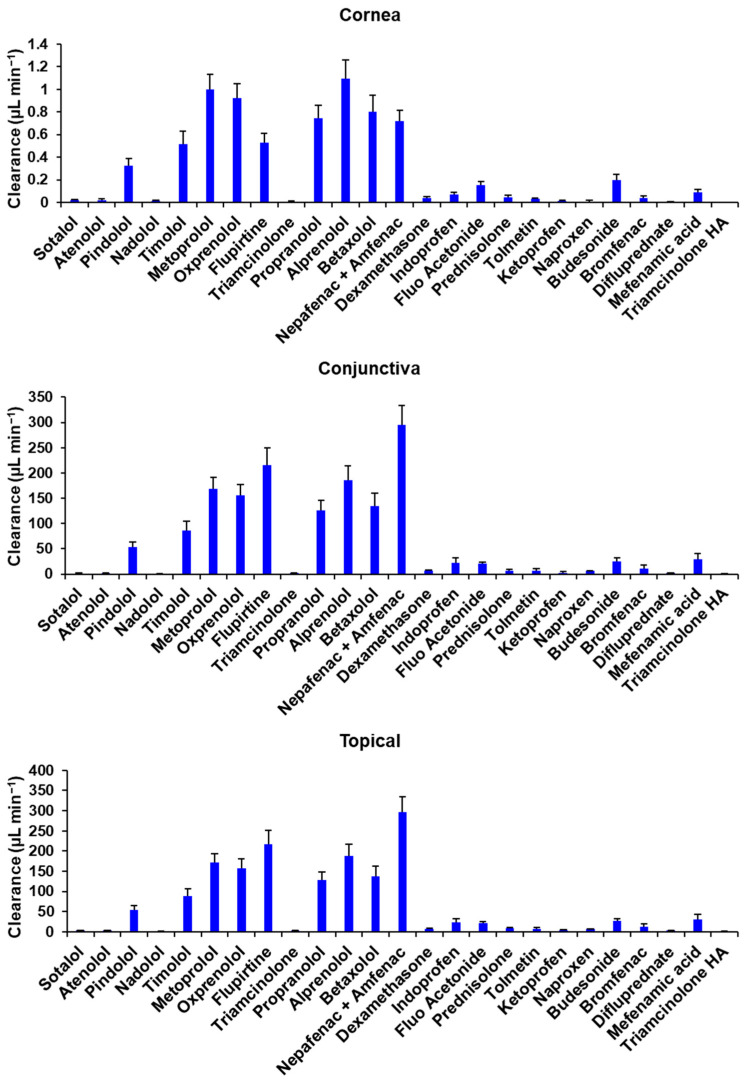
Calculated values of clearance through human cornea, human conjunctiva, and total topical clearance. Mean ± STD is reported for 5 estimates.

The regression equations (Table 5) used to estimate *P_app_* values through the conjunctiva had high coefficients of determination for all drug classes, and the values for clearance obtained are presented in Figure 5.

**Figure 6:**
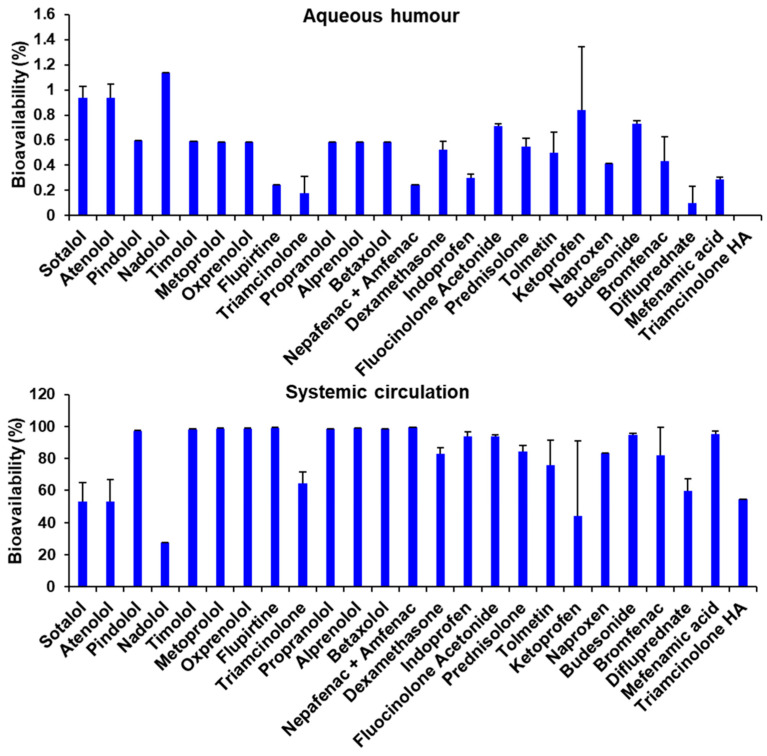
The estimated bioavailability values in aqueous humor and in systemic circulation for the human eye using porcine tissue permeability values. Mean ± STD is reported for 5 estimates.

The bioavailability values in aqueous humor and in systemic circulation are presented in Figure 6. The magnitude is close to what is expected for ocular bioavailability, but generally higher for conjunctiva based systemic bioavailability. The trends for different drugs did not follow what is anticipated based on drug lipophilicity and literature reports, consistent with the limitation of the bioavailability estimation methods in this study.

## 4. Discussion

Rabbit cornea has been widely used in ocular research, with the permeation behavior of many drugs well characterized, as summarized by Prausnitz and Noonan [45]. However, the porcine cornea holds important similarities with the human cornea, suggesting that it might be a more appropriate model to assess drug permeation. Van den Berghe, Guillet, and Compan [28] compared the central corneal thickness and number of layers of the epithelium of porcine, human, bovine, and rabbit corneas and indicated that porcine and human corneas are similar in this regard, while the bovine cornea is much thicker with more epithelial cell layers, and rabbit cornea is thinner with a similar number of cell layers in the corneal epithelium. Greiner et al. [46] suggested that human and porcine corneas had more comparable phosphate metabolism, relative to rabbit [47]. Porcine and rabbit corneas have significantly higher collagen fibrillar diameter, interfibrillar distance, and interlamellar distance compared to human cornea, but the magnitudes are of a similar order [48]. The presence of a Bowman’s layer in porcine cornea, previously an object of controversy among specialists [49], has also been demonstrated in more recent studies [48,50], marking another similarity with human, rabbit, and bovine corneas [51].

In this study, rabbit cornea showed higher transport and *P_app_* values than bovine and porcine corneas, which had values closer to each other. This finding might be related to the lower thickness of the rabbit cornea, which can deliver drugs to the anterior chamber faster than thicker tissues. For all tissues, permeability was higher for drugs with intermediate lipophilicity, mostly β-blockers, flupirtine, and nepafenac. Permeability might also be affected by drug ionization in the medium since charged molecules remain in the aqueous medium to a greater degree. By observing data through this prism, we can notice that the highest transported amounts were observed for drugs with a higher pKa, behaving as weak bases that tend to be ionized at pH 7.4. For most drugs, the bovine and porcine permeabilities were comparable. However, β-blockers, flupirtine, and nepafenac showed higher permeability through the bovine cornea. Differences have been reported in the conformation of collagen packaging between these two tissues, with porcine displaying more regular, approximately orthogonal layers and bovine displaying more randomly interwoven layers [52]. These differences might also have a role in the behavior of the cornea as a barrier since regular packaging may provide higher fiber density to the tissue, thereby increasing barrier capacity.

Tissue uptake behaved like cumulative transport, with the influence of the epithelium layer more noticeable. The lipophilic character of this layer increases the retention of lipophilic drugs, which was the case for all species. It is possible to notice that bovine and porcine corneas showed higher uptake of drugs with intermediate lipophilicity, while rabbit cornea showed higher uptake of drugs with higher lipophilicity. Rabbit cornea has a lower thickness than the other tissues, with this difference observed mainly for the hydrophilic stroma. Therefore, it is possible that in rabbit cornea, the lipophilic character of the epithelium is more prominent, thus favoring the uptake of lipophilic drugs per unit of tissue weight. On the other hand, for bovine and porcine corneas, the stroma might have a higher influence, allowing for the uptake of less lipophilic drugs.

A high correlation was found between *P_app_* and cumulative transport for all species. This correlation is expected since *P_app_* is obtained from the slope of the cumulative transport curve, and all cumulative transport data were normalized to the percent of the initial amount. The same degree of correlation was not observed between cumulative transport or *P_app_* and tissue uptake, with weak to no correlation observed for all species, which indicates the influence of different factors on these two phenomena.

When comparing species, a high correlation was also found for cumulative transport and *P_app_* between the species. A good to weak correlation was found between porcine or bovine and rabbit for tissue uptake, although the uptake across species was similar based on a slope close to 1. A high correlation was observed between bovine and porcine cornea for this parameter. It is important to note that bovine and porcine corneas showed not only a high correlation for the parameters measured but also that the correlation slopes were close to 1, indicating that the values observed were of the same order. Generally, the permeability differed between the species more than the tissue uptake, consistent with differences in tissue thickness.

*P_app_* and tissue uptake showed a good correlation with LogD_7_._4_ (Figure 4), which was the most relevant molecular descriptor indicated by modeling. The correlation followed a sigmoidal relationship, also reported by some authors [17,53,54]. In this relationship, the LogD_7_._4_ value at half-maximum was taken as a comparison parameter (Table 2) and showed that, for β-blockers and steroids, optimal LogD_7_._4_ values for tissue permeation were lower than those for uptake. NSAIDs, on the other hand, showed the opposite behavior of β-blockers and steroids, with higher half-maximum LogD_7_._4_ values for *P_app_* than for tissue uptake and higher maximum uptake for rabbit cornea. While permeability for the three drug classes and uptake for NSAIDs appeared to reach a more definite maximum in all species, tissue uptake of β-blockers in all species and corticosteroids in bovine cornea appeared to have more room for accumulation with a further increase in LogD beyond what was tested. The lack of saturation of corticosteroid uptake in the bovine eye might be due to the greater number of epithelial cell layers in this species. In general, the highest uptake and permeability were observed for β-blockers.

Data modeling using MLR provided models with a high degree (0.822 ≤ R ≤ 0.940) of correlation for all parameters tested. For the permeability and cumulative transport parameters, the best-fit models contained as x-variables the number of hydrogen bond sites, the nitrogen atom ratio in the molecule, the number of rotatable bonds, the molar volume, the index of refraction, and the LogD at a pH of 7.4. It is important to note that for the permeability parameter, although the apparent permeability coefficient (*P_app_*) was calculated, better models were obtained with flux as the y-variable.

For permeability and cumulative transport models, the number of hydrogen bond acceptor or donor sites is a variable frequently reported [55,56,57]. This parameter is directly related to the degree of polarity of the molecule, and, therefore, these sites can limit to a certain degree the permeation of a molecule through the barrier of the corneal epithelium, which is strongly lipophilic.

The nitrogen atom ratio in the molecule can also be a factor of strong influence on its permeability since these atoms can form polar groups in the molecule, participating in the formation of hydrogen bonds. Furthermore, nitrogen atoms are often present in these molecules as amine groups, which have a direct influence on the ionization state of the molecules at the pH of the medium. The ionization of molecules is a determining factor for their permeability not only through the cornea but also through any biological membrane [48].

The variable LogD at a pH of 7.4 was present in the most relevant models not only for cumulative transport and permeability but also for tissue uptake, being identified as the most relevant variable among those tested. Since LogD describes the octanol/water partition coefficient at experimental pH, this variable combines the influence of molecular structure and ionizable groups in its hydrophobicity, showing better experimental applicability than LogP in this context.

The refractive index is defined as the ratio between the speed of light in each substance and in a vacuum. The modification of the light speed when passing through a substance is due to the interaction between the electric field of light and the electronic cloud of the molecules. If the oscillation frequency of the incident light is like that of a particular atom, group, or molecule, the greater the interaction between them, the greater the refraction of light. Rocquefelte et al. [58] pointed out that the refractive index of a substance is closely related to its mass density, electron density, and the presence of chromophore groups and excitable electrons in the molecules. Several electron-dense groups are present in the molecules studied and contribute to the refraction of light since, in addition to absorbing radiation, these groups also enable greater attraction between drug molecules, such as nitrogen and oxygen heteroatoms, amine, carboxyl, carbonyl, and hydroxyl groups, as well as halogens.

Another factor that was shown to be relevant for the three tissues tested was the count of rotatable bonds in the molecules. Rotatable bonds comprise any single bond, not in a ring, bound to a nonterminal heavy (non-hydrogen and non-nitrogen) atom [59]. The influence of this variable has been described by some authors as either limiting or contributing to drug absorption, e.g., Veber et al. [59], Zakeri-Milani et al. [60], Iyer et al. [61], and Davis, Gerry, and Tan [62]. Either way, the presence of rotatable bonds can lead to conformational changes in the molecules, and these shape changes can determine the molecule’s ability to pass through or be retained in densely packed barriers such as corneal epithelium [63].

Modeling results have indicated the influence of molar volume on permeability through the cornea. This size-related descriptor has also been used [64,65] as a correction factor for permeability through other biological and artificial membranes, since size may be a determinant property for permeability limited by porous structures.

Data modeling for the tissue uptake parameter showed the relevance of different variables for the different tissues evaluated, although it is possible to highlight some similarities between them. First, it is possible to verify the influence of the presence of polar regions in the molecules, represented by the variables of hydrogen bond acceptor sites for bovine and the sum of hydrogen bond donor and acceptor sites in the rabbit cornea, as well as by the ratios of nitrogen and of heteroatoms in the molecules. The influence of polarity is also represented by the variable LogD at pH 7.4 in all the models obtained. Structural properties also had an influence on the uptake, as evidenced by the relevance of rotatable bonds and molecular weight. It is important to notice that, regarding size-related properties such as molar volume and molecular weight, the latter might account better for the presence of oxygen and nitrogen atoms since these atoms might increase the molecular weight without significantly altering the molar volume [66].

Bioavailability assessment represents a crucial step in drug feasibility evaluation. When considering ocular drug delivery, bioavailability is restricted by barriers present in the eye. For topical drug delivery to the anterior chamber, three main barriers may be considered: blinking and tear fluid drainage, cornea, and conjunctiva, each presenting unique absorption-limiting characteristics.

The conjunctival epithelium is composed of 2 to 3 stratified cell layers bound by tight junctions, but different from corneal epithelium, conjunctiva presents a higher density of pores ranging from 4.9 to 3.0 nm in diameter, thus permitting the paracellular absorption of hydrophilic and large molecules, according to Lawrence and Miller [67]. These structural properties make the conjunctiva more permeable than the cornea for many drug classes. However, the permeability properties of the conjunctiva depend on isolation and tissue mounting techniques, with poor isolation techniques resulting in the loss of integrity of the conjunctival barrier and the associated trans-tissue electrical resistance [68]. Thus, in vitro studies are inherently limited. Although a precise prediction of the in vivo pharmacokinetics of a drug will remain elusive, some useful information can be gathered from in vitro permeability data. Assuming that conjunctival permeability predominantly accounts for systemic availability while corneal permeability contributes predominantly to aqueous humor bioavailability, we estimated the bioavailability for various drugs in this study. Our estimates indicated that overall bioavailability in aqueous humor after corneal absorption would be limited, with values ranging from 0.00 to 1.13%. The range observed is related to corneal barrier properties and perm-selectivity, which allow restricted and differential permeability to different compounds. Low aqueous humor bioavailability is expected for most drugs administered topically due to corneal barrier properties discussed previously in this article. It is apparent that systemic availability did not follow the previously reported trend. A limitation of the present approach is that blink-induced rapid tear drainage was not considered. Additionally, nasal absorption contributes 50% or more to the systemic absorption of β-blockers [15]. Thus, nasal permeability and blink-induced drainage rates should be factored in to better estimate the systemic as well as topical bioavailability of ophthalmic drugs. Further, most literature-reported permeabilities for the conjunctiva may be overestimates due to improper tissue isolation, magnifying the conjunctival clearance of drugs estimated in this study.

For β-blockers, diffusion and partition coefficients in the conjunctiva are reportedly higher than in the cornea [69]. Results indicated that systemic bioavailability would be more than 96% for β-blockers, except for sotalol, atenolol, and nadolol, hydrophilic β-blockers, which would be more susceptible to tear drainage but less permeable. This result agrees with the ones by Lee, Kompella, and Lee [15], in which the authors describe lower systemic bioavailability for hydrophilic β-blockers such as atenolol (41%). The authors also determined that nasal drainage contributes more to systemic absorption than the conjunctival pathway for systemic absorption. Corticosteroids, however, being predominantly lipophilic, showed possibly more limited conjunctival absorption than β-blockers, reflected mainly in triamcinolone, difluprednate, and triamcinolone hexacetonide. It is important to note that in rabbit conjunctival epithelial cells, the presence of p-glycoprotein efflux pumps was reported, which might hinder the absorption of lipophilic drugs [70]. Such mechanisms are described also for human and rabbit corneas [71] and might also be present in porcine cornea, thus explaining the lower slope observed for this drug class in the model obtained (Table 5). NSAIDs, on the other hand, would benefit from the presence of a sodium-dependent monocarboxylate transport process in the mucosal side of the conjunctiva [72], which would facilitate absorption. The influence of this mechanism might be reflected in the high model slope and bioavailability values found for this drug class, with ketoprofen being the only NSAID with an estimated systemic bioavailability lower than 80%.

## 5. Conclusions

Ex vivo corneal models for drug permeability are relevant in the preclinical stages of drug evaluation to identify drug candidates or formulations with superior permeability and to predict drug bioavailability in vivo. Different species have been used to evaluate the permeation of drugs through the cornea, including rabbit, porcine, and bovine. The present study compared the permeability of twenty-five drugs across the corneas of these three species and developed predictive equations as well as interspecies correlations for drug permeability and tissue uptake. This study established a single equation to predict corneal drug delivery in multiple species based on tissue thickness and drug properties such as lipophilicity and polar intermolecular interactions. Such equations help explain species differences in drug permeability and delivery.

A high correlation was observed between bovine, porcine, and rabbit corneas for permeability, with the permeability being comparable for bovine and porcine eyes and higher for the rabbit cornea. Permeability differences between species could be explained in part by differences in corneal thickness. Although tissue uptakes in all three species were similar, a high correlation was observed for tissue uptake between bovine and porcine corneas. The correlations between the properties observed in bovine and porcine corneas indicate that bovine cornea is acceptable for drug or formulation screening since it can provide data like porcine tissues, which are reportedly similar to human corneal tissue.

Estimation of drug bioavailability using ex vivo data indicated that topical absorption into aqueous humor would be limited for all drugs considered, with most of the dose being absorbed into the systemic circulation. Although the order of magnitude of bioavailability in aqueous humor may be comparable to literature-reported values, the trends may not be as reported. There is room to improve bioavailability predictions.

Bovine cornea has significantly higher thickness [36] than porcine and rabbit corneas, with more cell layers in the epithelium [28]. Stroma is also responsible for the higher thickness of bovine cornea [33]. Bovine cornea was established as a model for ocular irritancy by Gautheron et al. in 1992 [73], and in 1994 [74], it was assessed as a model for permeability by the same group. However, since then, bovine cornea has been used mainly for the evaluation of ocular irritancy. Thus, this work presents an important contribution to the literature since it evaluated bovine corneal permeability and the uptake of several drugs.

Future studies will evaluate in vivo delivery of the cassette, the effect of the cassette on barrier integrity, and enhance mathematical models to predict in vivo delivery based on in vitro delivery.

## Figures and Tables

**Table 1:** Corneal thickness reported in the literature for each tissue tested.

Corneal Thickness (µm)		
Bovine	Porcine	Rabbit
800 [27]	950 [28]	480 [28]
1530 [28]	1188 [29]	370 [27]
1015 [30]	955 [31]	381 [32]
1024 [33]	850 [34]	500 [35]
1160 [36]	851 [37]	422 [38]
1105.8 *	958.8 *	430.6 *

* Average value.

**Table 2:** LogD_7.4_ at half-maximum of the sigmoidal curves obtained for permeability and tissue uptake.

Tissue	Parameter	LogD7.4 at Half-Maximum		
		β-Blockers	NSAIDs	Corticosteroids
Bovine	Papp (cm s^−1^)	−0.3521	0.5118	2.145
	Tissue uptake (% g^−1^)	0.2783	0.5068	5.61
Porcine	Papp (cm s^−1^)	−0.3327	0.4692	2.202
	Tissue uptake (% g^−1^)	1.093	0.3586	2.43
Rabbit	Papp (cm s^−1^)	−0.3578	0.6092	2.023
	Tissue uptake (% g^−1^)	1.758	0.2055	2.204

**Table 3:** Best-fit predictive models obtained for each tissue and each parameter evaluated.

Parameter	Tissue	Unit	Coefficients	R^2^	Q^2^
Permeability	Bovine	Flux (log % s^−1^/cm^2^)	(8.312) + (−0.236·HBA) + (7.254·NR) + (−7.192·IR) + (0.348·LogD7.4)	0.87	0.735
		Papp (log cm s^−1^)	(−5.84) + (−0.101·HBDA) + (0.141·RB) + (3.994·NR) + (0.274·LogD7.4)	0.812	0.632
	Porcine	Flux (log % s^−1^/cm^2^)	(11.882) + (−0.319·HBA) + (10.175·NR) + (−9.504·IR) + (0.503·LogD7.4)	0.857	0.704
		Papp (log cm s^−1^)	(−24.374) + (−0.329·HBDA) + (8.346·Log MV) + (14.591·NR) + (0.227·LogD7.4)	0.722	0.462
	Rabbit	Flux (log % s^−1^/cm^2^)	(10.143) + (−0.279·HBA) + (7.64·NR) + (−8.174·IR) + (0.47·LogD7.4)	0.884	0.748
		Papp (log cm s^−1^)	(−5.496) + (−0.061·HBDA) + (0.11·RB) + (2.308·NR) + (0.245·LogD7.4)	0.781	0.537
Cumulative transport	Bovine	Amount (%)	(179.182) + (−2.468·HBA) + (81.565·NR) + (−106.291·IR) + (3.247·LogD7.4)	0.764	0.529
		Amount (%)	(−6.868) + (2.251·RB) + (2.282·LogD7.4)	0.676	0.492
	Porcine	Amount (%)	(142.4) + (−1.956·HBA) + (57.287·NR) + (−84.66·IR) + (2.559·LogD7.4)	0.75	0.522
		Amount (%)	(−6.025) + (1.754·RB) + (1.857·LogD7.4)	0.691	0.5
	Rabbit	Amount (%)	(253.788) + (−3.628·HBA) + (110.828·NR) + (−149.001·IR) + (5.481·LogD7.4)	0.801	0.617
		Amount (%)	(−7.379) + (3.048·RB) + (4.057·LogD7.4)	0.675	0.493
Tissue uptake	Bovine	Amount (% g^−1^)	(0.763) + (−0.293·HBA) + (0.145·RB) + (5.139·HR) + (0.426·LogD7.4)	0.863	0.69
	Porcine	Amount (% g^−1^)	(7.694) + (−2.834·Log MW) + (0.118·RB) + (0.419·LogD7.4)	0.728	0.562
	Rabbit	Amount (% g^−1^)	(2.23) + (−0.146·HBDA) + (0.041·RB) + (2.376·NR) + (0.216·LogD7.4)	0.807	0.657

HBA: hydrogen bond acceptors; HBDA: hyd. bond donors + acceptors; NR: N ratio; RB: rotatable bonds; HR: heteroatom ratio; IR: index of refraction; LogD: LogD at pH = 7.4; Log MV: log molar volume; Log MW: log molecular weight.

**Table 4:** Best-fit predictive models obtained for each parameter evaluated, considering tissue thickness.

Parameter	Unit	Model	R^2^	Q^2^
Flux	−log %·s^−1^/cm^2^	(3.474) + (3.833·TT) + (0.138·HBDA) + (−0.135·RB) + (−5.07·NR) + (−0.375·LogD)	0.781	0.719
Flux	−log %·s^−1^/cm^2^	(−10.432) + (3.833·TT) + (0.279·HBA) + (−8.357·NR) + (8.29·IR) + (−0.441·LogD)	0.826	0.780
P_app_	−log cm s^−1^	(5.41) + (7.257·TT) + (0.086·HBDA) + (−0.151·RB) + (−3.872·NR) + (−0.315·LogD)	0.691	0.608
P_app_	−log cm s^−1^	(−9.001) + (7.256·TT) + (0.207·HBA) + (−7.687·NR) + (8.548·IR) + (−0.374·LogD)	0.716	0.650
Cumulative transport	%	(6.118) + (−84.079·TT) + (−1.383·HBA) + (2.093·RB) + (25.45·NR) + (3.029·LogD)	0.700	0.610
Cumulative transport	%	(198.783) + (−84.079·TT) + (−2.685·HBA) + (83.227·NR) + (−113.318·IR) + (3.763·LogD)	0.734	0.662
Tissue uptake	log % g^−1^	(1.422) + (−2.235·TT) + (−0.303·HBA) + (0.105·RB) + (4.442·HR) + (0.387·LogD)	0.750	0.684
Tissue uptake	log % g^−1^	(1.853) + (−2.235·TT) + (−0.13·HBDA) + (0.097·RB) + (2.615·NR) + (0.308·LogD)	0.748	0.678

TT: tissue thickness; HBA: hydrogen bond acceptors; HBDA: hyd. bond donors + acceptors; NR: N ratio; RB: rotatable bonds; HR: heteroatom ratio; IR: index of refraction; LogD: LogD at pH = 7.4.

**Table 5:** Models used to estimate conjunctival Papp values for porcine cornea.

Drug Class	R^2^	Model
β-blockers	0.934	*P_app_*_Conjunctiva_ = (10.141·*P_app_*_Cornea_) + (−0.00000267)
NSAIDs	0.979	*P_app_*_Conjunctiva_ = (24.645·*P_app_*_Cornea_) + (−0.00000633)
Corticosteroids	0.999	*P_app_*_Conjunctiva_ = (7.150·*P^app^*_Cornea_) + (0.00000136)

## Data Availability

The data presented in this study are available within the article or in the Supplementary Material.

## Supplementary Materials

The following supporting information can be downloaded at: https://www.mdpi.com/article/10.3390/pharmaceutics15061646/s1, Table S1: Physicochemical properties of drugs used in building quantitative structure-permeability relationships for bovine, porcine, and rabbit corneas.
