# Supplementary material for: Corneal Permeability and Uptake of Twenty-Five Drugs: Species Comparison and Quantitative Structure–Permeability Relationships
Source: Pharmaceutics. 2023 Jun 2;15(6):1646. doi: 10.3390/pharmaceutics15061646 (PMC10302615; doi:10.3390/pharmaceutics15061646)
Supplement: Supplementary file 1 [file pharmaceutics-15-01646-s001.zip › pharmaceutics-2387555-supplementary.pdf]

Manuscript title: Corneal Permeability and Uptake of Twenty-Five Drugs: Species Comparison and Quantitative Structure–Permeability Relationships

Authors: Cleildo P. Santana, Brock A. Matter, Madhoosudan A. Patil, Armando Silva-Cunha, and Uday B. Kompella

Journal: Pharmaceuticss

Supplementary Table S1: Physicochemical properties of drugs used in building quantitative structure-permeability relationships for bovine, porcine, and rabbit corneas.

Drug Name	Molecular Weight	Log Molecular Weight	Hydrogen Bond Donors	Hydrogen Bond Acceptors	Hyd. Bond Donors + Acceptors	TPSA	Rotatable Bonds	C Ratio	N Ratio	NO Ratio	Hetero Ratio	Halogen Ratio	Number of Rings	Aromatic Rings
Alprenolol	249.35	2.40	2.00	3.00	5.00	41.49	8.00	0.83	0.06	0.17	0.17	0.00	1.00	1.00
Atenolol	266.34	2.43	4.00	5.00	9.00	84.58	8.00	0.74	0.11	0.26	0.26	0.00	1.00	1.00
Betaxolol	307.43	2.49	2.00	4.00	6.00	50.72	11.00	0.82	0.05	0.18	0.18	0.00	2.00	1.00
Bromfenac	334.16	2.52	3.00	4.00	7.00	80.39	4.00	0.75	0.05	0.20	0.20	0.05	2.00	2.00
Budesonide	430.53	2.63	2.00	6.00	8.00	93.06	4.00	0.81	0.00	0.19	0.19	0.00	5.00	0.00
Dexamethasone	392.46	2.59	3.00	5.00	8.00	94.83	2.00	0.79	0.00	0.18	0.18	0.04	4.00	0.00
Difluprednate	508.55	2.71	1.00	7.00	8.00	106.97	8.00	0.75	0.00	0.19	0.19	0.06	4.00	0.00
Fluocinolone Acetonide	452.49	2.66	2.00	6.00	8.00	93.06	2.00	0.75	0.00	0.19	0.19	0.06	5.00	0.00
Flupirtine	304.32	2.48	4.00	6.00	10.00	89.27	6.00	0.68	0.18	0.27	0.27	0.05	2.00	2.00
Indoprofen	281.31	2.45	1.00	4.00	5.00	57.61	3.00	0.81	0.05	0.19	0.19	0.00	3.00	2.00
Ketoprofen	254.28	2.41	1.00	3.00	4.00	54.37	4.00	0.84	0.00	0.16	0.16	0.00	2.00	2.00
Mefenamic Acid	241.29	2.38	2.00	3.00	5.00	49.33	3.00	0.83	0.06	0.17	0.17	0.00	2.00	2.00
Metoprolol	267.36	2.43	2.00	4.00	6.00	50.72	9.00	0.79	0.05	0.21	0.21	0.00	1.00	1.00
Nadolol	309.40	2.49	4.00	5.00	9.00	81.95	6.00	0.77	0.05	0.23	0.23	0.00	2.00	1.00
Naproxen	230.26	2.36	1.00	3.00	4.00	46.53	3.00	0.82	0.00	0.18	0.18	0.00	2.00	2.00
Nepafenac	254.28	2.41	4.00	4.00	8.00	86.18	4.00	0.79	0.11	0.21	0.21	0.00	2.00	2.00
Oxprenolol	265.35	2.42	2.00	4.00	6.00	50.72	9.00	0.79	0.05	0.21	0.21	0.00	1.00	1.00
Pindolol	248.32	2.40	3.00	4.00	7.00	57.28	6.00	0.78	0.11	0.22	0.22	0.00	2.00	2.00
Prednislone	360.44	2.56	3.00	5.00	8.00	94.83	2.00	0.81	0.00	0.19	0.19	0.00	4.00	0.00
Propranolol	259.34	2.41	2.00	3.00	5.00	41.49	6.00	0.84	0.05	0.16	0.16	0.00	2.00	2.00
Sotalol	272.36	2.44	3.00	5.00	8.00	86.81	6.00	0.67	0.11	0.28	0.33	0.00	1.00	1.00
Timolol	316.42	2.50	2.00	7.00	9.00	107.98	7.00	0.62	0.19	0.33	0.38	0.00	2.00	1.00
Tolmetin	257.28	2.41	1.00	4.00	5.00	59.30	4.00	0.79	0.05	0.21	0.21	0.00	2.00	2.00
Triamcinolone	394.43	2.60	4.00	6.00	10.00	115.06	2.00	0.75	0.00	0.21	0.21	0.04	4.00	0.00
Triamcinolone Hexacetonide	532.64	2.73	1.00	7.00	8.00	99.13	6.00	0.79	0.00	0.18	0.18	0.03	5.00	0.00

Drug Name	Number of Rings (size 5)	Number of Rings (size 6)	Log(BCF)	Parachor	Index of Refraction	Surface Tension	Density	Polarizability	Molar Volume	Log Molar Volume
Alprenolol	0.00	1.00	1.96	607.71	1.52	36.37	1.01	29.75	247.46	2.39
Atenolol	0.00	1.00	-0.16	613.02	1.54	45.02	1.13	29.44	236.66	2.37
Betaxolol	0.00	1.00	1.81	730.89	1.53	41.48	1.07	35.25	287.99	2.46
Bromfenac	0.00	2.00	1.84	601.63	1.66	63.11	1.57	31.35	213.46	2.33
Budesonide	2.00	3.00	2.16	917.26	1.59	55.25	1.28	45.14	336.45	2.53
Dexamethasone	1.00	3.00	1.19	812.36	1.59	56.57	1.32	39.74	296.21	2.47
Difluprednate	1.00	3.00	2.56	1038.86	1.54	48.96	1.29	49.22	392.74	2.59
Fluocinolone Acetonide	2.00	3.00	1.48	890.07	1.58	52.71	1.37	43.41	330.34	2.52
Flupirtine	0.00	2.00	1.28	628.61	1.66	60.65	1.35	33.03	225.26	2.35
Indoprofen	1.00	2.00	1.88	596.36	1.64	59.23	1.31	30.81	214.97	2.33
Ketoprofen	0.00	2.00	1.91	563.75	1.59	49.77	1.20	28.46	212.25	2.33
Mefenamic Acid	0.00	2.00	3.82	538.03	1.64	51.80	1.20	28.63	200.55	2.30
Metoprolol	0.00	1.00	1.13	638.37	1.51	37.06	1.03	30.55	258.73	2.41
Nadolol	0.00	2.00	0.75	679.52	1.57	46.64	1.19	33.99	260.03	2.42
Naproxen	0.00	2.00	2.05	504.61	1.61	47.47	1.20	26.37	192.25	2.28
Nepafenac	0.00	2.00	0.32	563.44	1.64	58.83	1.25	29.10	203.44	2.31
Oxprenolol	0.00	1.00	1.51	627.57	1.51	36.60	1.04	30.45	255.15	2.41
Pindolol	1.00	1.00	1.27	565.76	1.60	47.52	1.15	29.11	215.48	2.33
Prednislone	1.00	3.00	0.91	766.87	1.61	60.74	1.31	37.85	274.70	2.44
Propranolol	0.00	2.00	2.12	606.16	1.58	42.67	1.09	31.31	237.16	2.38
Sotalol	0.00	1.00	0.01	586.04	1.57	50.62	1.24	28.60	219.71	2.34
Timolol	1.00	1.00	0.29	695.59	1.55	52.46	1.22	32.57	258.46	2.41
Tolmetin	1.00	1.00	0.95	560.00	1.58	43.56	1.18	28.83	217.98	2.34
Triamcinolone	1.00	3.00	0.40	789.31	1.62	65.58	1.42	38.51	277.36	2.44
Triamcinolone Hexacetonide	2.00	3.00	3.85	1128.17	1.56	48.59	1.25	54.52	427.30	2.63

Drug Name	Molar Refractivity	LogS	LogP	$LogD_{7.2}$	$LogD_{7.3}$	$LogD_{7.4}$	$LogD_{7.5}$	$LogD_{7.6}$	pKa(Acid)
Alprenolol	75.05	0.42	2.78	0.51	0.59	0.68	0.77	0.86	13.88
Atenolol	74.26	0.57	0.24	-2.03	-1.94	-1.85	-1.76	-1.67	13.88
Betaxolol	88.92	-0.13	2.87	0.59	0.68	0.77	0.86	0.95	13.89
Bromfenac	79.09	-0.43	3.26	0.36	0.28	0.20	0.12	0.05	4.07
Budesonide	113.86	-4.00	3.02	3.02	3.02	3.02	3.02	3.02	12.87
Dexamethasone	100.24	-3.77	1.92	1.92	1.92	1.92	1.92	1.92	12.13
Difluprednate	124.16	-4.45	3.17	3.17	3.17	3.17	3.17	3.17	12.56
Fluocinolone Acetonide	109.51	-3.95	2.39	2.39	2.39	2.39	2.39	2.39	12.78
Flupirtine	83.32	-3.47	2.47	2.47	2.47	2.47	2.47	2.47	12.19
Indoprofen	77.71	-0.98	3.02	0.32	0.23	0.14	0.05	-0.03	4.39
Ketoprofen	71.80	-0.39	3.12	0.22	0.13	0.05	-0.02	-0.10	4.23
Mefenamic Acid	72.21	-0.73	5.00	2.13	2.08	2.04	2.01	1.98	3.73
Metoprolol	77.07	0.57	1.81	-0.47	-0.38	-0.29	-0.20	-0.11	13.89
Nadolol	85.75	0.51	1.24	-1.03	-0.95	-0.86	-0.77	-0.68	13.91
Naproxen	66.52	-1.32	2.98	0.64	0.54	0.45	0.36	0.27	4.84
Nepafenac	73.40	-2.85	1.38	1.38	1.38	1.38	1.38	1.38	16.09
Oxprenolol	76.81	0.58	2.29	0.01	0.10	0.18	0.27	0.37	13.89
Pindolol	73.42	0.29	1.86	-0.49	-0.41	-0.32	-0.23	-0.14	13.94
Prednislone	95.48	-3.26	1.66	1.66	1.66	1.66	1.66	1.66	12.46
Propranolol	78.99	-0.30	3.26	0.97	1.06	1.15	1.24	1.33	13.84
Sotalol	72.14	0.56	0.38	-1.71	-1.63	-1.54	-1.45	-1.37	8.46
Timolol	82.16	0.50	1.53	-0.53	-0.43	-0.34	-0.25	-0.15	13.38
Tolmetin	72.72	-0.19	2.40	-0.51	-0.59	-0.67	-0.75	-0.82	4.22
Triamcinolone	97.14	-3.40	0.92	0.92	0.92	0.92	0.92	0.92	11.57
Triamcinolone Hexacetonide	137.53	-5.15	4.70	4.70	4.70	4.70	4.70	4.70	13.09
